# Looking beyond the Skin: Pathophysiology of Cardiovascular Comorbidity in Psoriasis and the Protective Role of Biologics

**DOI:** 10.3390/ph15091101

**Published:** 2022-09-03

**Authors:** Isabel Andújar, Juan V. Esplugues, Patricia García-Martínez

**Affiliations:** 1 Departamento de Farmacología, Universidad de Valencia, 46010 Valencia, Spain; 2 FISABIO—Hospital Universitario Dr. Peset, 46017 Valencia, Spain; 3 Centro de Investigación Biomédica en Red de Enfermedades Hepáticas y Digestivas (CIBERehd), 46010 Valencia, Spain

**Keywords:** psoriasis, biologics, atherosclerosis, cardiovascular, anti-TNFα, anti-IL17, anti-IL12, anti-IL23

## Abstract

Psoriasis is a chronic systemic inflammatory disease associated with a higher incidence of cardiovascular disease, especially in patients with moderate to severe psoriasis. It has been estimated that severe psoriasis confers a 25% increase in relative risk of cardiovascular disease, regardless of traditional risk factors. Although the underlying pathogenic mechanisms relating psoriasis to increased cardiovascular risk are not clear, atherosclerosis is emerging as a possible link between skin and vascular affection. The hypothesis that the inflammatory cascade activated in psoriasis contributes to the atherosclerotic process provides the underlying basis to suggest that an anti-inflammatory therapy that improved atherosclerosis would also reduce the risk of MACEs. In this sense, the introduction of biological drugs which specifically target cytokines implicated in the inflammatory cascade have increased the expectations of control over the cardiovascular comorbidity present in psoriasis patients, however, their role in vascular damage processes remains controversial. The aim of this paper is to review the mechanistic link between psoriasis and cardiovascular disease development, as well as analyzing which of the biological treatments could also reduce the cardiovascular risk in these patients, fueling a growing debate on the modification of the general algorithm of treatment.

## 1. Introduction

Psoriasis is a chronic inflammatory disease whose characteristic manifestation is hyperplasia of the epidermis in the form of erythematous and scaling lesions on the skin as the ultimate consequence of hyperproliferation of epidermal keratinocytes [1]. Currently, it is defined as a polygenic and immune-mediated disease of unknown cause, influenced by environmental and psychosomatic factors [2]. Similar to most autoimmune or immune-mediated inflammatory pathologies, psoriasis fluctuates between periods of remission and exacerbation [2]. In terms of its prevalence, which varies greatly depending on the geographical area, it is estimated that it affects between 1% and 3% of the world population [3], and it is one of the few non-communicable diseases that the World Health Organization has identified as a major health problem.

Many studies over the last decade have warned that psoriasis is not an exclusive skin disease, but rather that it should be viewed as a systemic disease [4]. There is evidence that the perpetuation of the inflammatory environment spreads beyond the immediate vicinity of skin lesions. As the pathogenesis of the disease progresses, the increase in proinflammatory markers takes place not only at the skin lesions, but also in the blood, evidencing its systemic nature. The serum levels of multiple cytokines, including TNFα, IFNγ, IL-6, IL-8, IL-12, IL-17A and IL-18, is elevated compared to the concentrations found in healthy individuals [5,6,7]. Moreover, the accumulation of neutrophils in psoriatic plaques and microabscesses is accompanied by an increase in these cells in the circulation [8,9], together with findings of subclinical inflammation in the liver, joints, and tendons, accompanied by a significant increase in vascular and subcutaneous inflammation [10,11]. In fact, approximately 73% of patients with psoriasis have a concomitant disease [12], presenting a higher prevalence of psoriatic arthritis, inflammatory bowel disease [13,14]—both of them explained by their common pathogenesis with psoriasis [1,2]—diabetes mellitus, obesity, metabolic syndrome (abdominal obesity, high blood pressure, hyperglycemia, and atherogenic dyslipidemia), cardiovascular diseases [15,16,17,18], or non-alcoholic fatty liver [19], among others.

The association between psoriasis and a higher incidence of cardiovascular disease (CVD) has been known for decades [15,16,17,18,20,21,22]. Although there is still some controversy about the milder forms of the disease [23,24,25], a higher prevalence of myocardial infarction [23,26], venous thromboembolism [27], stroke [27,28], ischemic heart disease, cerebrovascular accidents [29], and a substantial increase in cardiovascular mortality [26,30] has been observed in patients with moderate to severe psoriasis. It has been estimated that severe psoriasis confers a 25% increase in the relative risk of CVD, regardless of traditional risk factors such as hyperlipidemia, smoking and obesity [31,32,33,34,35,36]; and a 6.2% additional absolute risk of experiencing a major adverse cardiovascular event (MACE) (e.g., myocardial infarction, stroke, cardiovascular death) within 10 years of diagnosis, compared to the healthy population [15,37].

In recent years, studies have been carried out with the aim of finding the pathways and/or cells responsible for the connection between psoriasis, cardiovascular risk factors and CVD. Although the underlying pathogenic mechanisms are not clear, the high prevalence of atherosclerosis [21] and the findings of subclinical atherosclerosis in these patients [38,39], even in those with a mild degree of skin involvement [10,11,39], suggest that this phenomenon could be the beginning of the bridge between skin and vascular affection, since atherosclerosis is also a key process in the development of CVD [40,41,42] and rupture of the atherosclerotic plaque is the key event leading to an acute MI.

The aim of this review is to present a thorough analysis of the data available to date regarding the mechanistic link between psoriasis and cardiovascular disease development, as well as analyzing which of the biological treatments available to date for psoriasis could also provide increased prevention of the development of these cardiovascular disorders.

## 2. Link between Atherosclerosis and Psoriasis

Atherosclerosis is a phenomenon driven by inflammation [43,44], which is why more than a decade ago the inflammatory character of psoriasis was proposed as a link between the skin condition and the development of atherosclerosis [45]. The most supported mechanistic hypothesis to explain the ability of psoriasis to act as an independent cardiovascular risk factor is commonly known as the *psoriatic march*. It suggests that the sum of environmental factors that trigger psoriasis (e.g., infection, tobacco, etc.), together with chronic systemic inflammation and the presence of concomitant diseases results in the development of insulin resistance. This insulin resistance subsequently induces endothelial dysfunction of the blood vessels, which leads to their hardening and the formation of atherosclerosis, that could ultimately lead to a cardiovascular event [46,47]. In turn, the three components of the psoriatic march (environmental factors, chronic systemic inflammation and concomitant diseases), through shared mechanisms and their inflammatory base, could feed off each other [1]. This concept is consistent with the fact that the persistent inflammation involved in psoriasis goes beyond the skin and is capable of involving multiple systemic mechanisms that make it an independent cardiovascular risk factor.

Together with the classic markers of inflammation, multiple studies have shown the clinical presence of insulin resistance in psoriasis patients [48,49], with its consequent endothelial dysfunction [50,51,52,53], and an increase in adipokines levels to similar of those of prediabetic individuals [48,49]. Insulin resistance is a prominent component of cardiovascular disorders due to its involvement in the generation of endothelial dysfunction [54], since insulin plays a relevant role in vascular homeostasis [55,56]. The perpetuation of inflammation experienced by patients with psoriasis gives rise to cytokines, adipokines and angiogenic factors that, generated in excess, are capable of acting as insulin antagonists [55,56,57,58,59], thus causing insulin resistance. In this sense, TNFα and IL-6 are the main agents responsible for this effect [57,58]. Adipokines in general and leptin in particular, in addition to hindering insulin functions, can promote atherosclerosis through direct immunomodulation in vascular tissue [58,59] and, in turn, further promote the expression of proinflammatory cytokines [60]. Elevated leptin in psoriasis patients has been shown to correlate with severity of skin disease and indices of subclinical atherosclerosis. The dysfunction of the endothelium caused by insulin resistance translates into an increase in its permeability to lipid particles and extravasation of leukocytes, thus facilitating the formation of atherosclerotic plaques which, in turn, contribute to the release of more inflammatory mediators and matrix metalloproteinases that perpetuate inflammation [61].

Together with insulin resistance, these patients also present lipid abnormalities, both quantitative and functional [62,63]. The mean levels of oxidized LDL (oxLDL) in patients with psoriasis are significantly higher than those of healthy controls [64], which demonstrates that this dyslipidemia detected in psoriatic patients is atherogenic. Lipid abnormalities also include impaired reverse cholesterol transport capacity of HDL [63] which, together with chronic inflammation, accelerate atherosclerotic vascular disease [60].

A third direct mechanism proposed linking psoriasis and the development of atherosclerosis is the generation of epicardial adipose tissue, since it has been demonstrated that its presence is significantly increased in patients with psoriasis, being an additional source of visceral fat deposition and inflammatory cytokines, that are associated with coronary artery disease [60].

Once the atherosclerotic layer is established, there are several factors that can promote the transition from its stable phenotype to an unstable one [61,65,66] and, among them, the inflammatory environment, capable of activating matrix metalloproteins and cell apoptosis or necrosis, contributing to plaque destabilization [67,68].

Consequently, the inflammatory condition that surrounds moderate–severe psoriasis is a primary factor both in the development of atherosclerotic plaques and in their subsequent destabilization, increasing the risk of rupture and formation of thrombotic processes, which can ultimately lead to cardiovascular events such as myocardial infarction and stroke.

### Atherosclerosis and Psoriasis: Shared Mechanistic Pathways

The molecular mechanisms and proinflammatory cytokine profile of psoriatic lesions are remarkably similar to those of atherosclerotic vascular lesions, with a comparable inflammatory infiltrate of T cells, macrophages, and monocytes [43,46,69,70]. The first study that showed the relationship between psoriasis, systemic inflammation and atherosclerosis, highlighted the altered endothelial function and the extravasation of leukocytes, mainly T lymphocytes, as an early step shared in the process of plaque formation both in atherosclerosis and in psoriasis [45]. In atherosclerosis, endothelial activation in the areas where nascent arterial plaques are located, promote the extravasation of monocytes and lymphocytes and the subsequent release of IL-12 and IL-23 by macrophages and dendritic cells. Differentiated Th1 cells further promote atherosclerotic plaque growth, while Th17 cells promote vessel formation and intraplaque hemorrhage [70]. All these T cell subtypes involved in the pathogenesis of atherosclerosis are also involved in psoriasis [43,46,69,70,71,72,73], with the Th1 [71,72,73] and Th17 lymphocytes [74,75] being the ones that have the predominant role in both processes. Consequently, the release of inflammatory mediators such as TNFα, IFNγ and interleukins IL-1β, IL-22, IL-17 [69,76], characteristic of Th1 and Th17, are found increased in both affections [71,72,73].

More recent studies have revealed the importance of the activation of the Th17 pathway and of IL-17 itself, specifically the IL-17A subtype, in the context of psoriatic and atherosclerotic inflammation, relating the overexpression of this cytokine in skin lesions to the existence of a proatherogenic state in the psoriatic patient [43]. Elevated levels of IL-17 can lead to further weakening of the fibrous cap, with subsequent plaque rupture and therefore risk of myocardial infarction [70]. Increasing knowledge suggests that the IL-23-activated Th17 pathway may be the main link between the cutaneous and metabolic manifestations of psoriasis [77].

The relevant role of the different blood cells is another of the characteristics shared by psoriasis and atherosclerosis. In plaque psoriasis, intraepidermal accumulations of neutrophils are formed [8,78]. These play a crucial role in the development of psoriasis [8,79]: their number, functions and phenotype are altered and can accumulate in localized areas of the stratum corneum (Munro microabscesses) [8,78]. Their activity after stimulation by IL-17A plays a fundamental role in the perpetuation of inflammation [80]. On the other hand, in atherosclerosis these cells are equally important: neutrophils interact with the damaged endothelium, they secrete chemotactic mediators which increase leukocyte recruitment, and they stimulate the development of macrophages to foam cells, the specific cell type that leads to atherosclerosis and that can predict endothelial dysfunction independently [60,81]. The presence of neutrophils in developing atherosclerotic plaques has been demonstrated not only in mouse models [82,83], but also in human atherosclerotic lesions [84], and its localization in occlusive thrombi suggests a role in atherosclerotic progression [85].

As to monocytes and macrophages, their participation has been demonstrated in both diseases: they are frequently detected in psoriatic lesions [79,86] actively contributing to the spread and exacerbation of the disease [87]; and they carry out processes such as plaque necrosis and thinning of a protective fibrous cap that promote atherosclerotic progression [88,89]. However, they can also carry out functions that can suppress plaque progression and promote its regression, depending on their phenotype [89].

Finally, platelets also seem to actively contribute to both pathologies. Their involvement in immune and inflammatory processes is increasingly being recognized. In fact, patients with psoriasis have elevated levels of platelet activation markers (spontaneous platelet hyperaggregability, mean platelet volume, plasma levels of P-selectin) [90,91]. Moreover, the combination of various indices of platelet activation helps predict the severity of psoriasis [90]. Mechanistically, activated platelets are believed to facilitate leukocyte extravasation [92] and this ability is linked to atherosclerosis [93,94]. In addition, their clinical relevance is evident, since platelet-inhibiting drugs are effective in the treatment and prevention of acute arterial thromboembolic episodes [95].

The identification of the similarities and shared mechanisms between the formation of the psoriatic plaque and the atherosclerotic plaque shows why both conditions feed off each other and frequently coexist in the same individual; but whether vascular involvement is a consequence of the skin process or an accompanying manifestation of the disease is a matter of debate.

## 3. Biologics in the Treatment of Psoriasis. Do They Also Address the Increased Cardiovascular Risk?

The hypothesis that the inflammatory cascade activated in psoriasis contributes to the atherosclerotic process has laid the groundwork to expect that anti-inflammatory therapy could also improve atherosclerosis and reduce the risk of MACEs. Both phototherapy and topical treatments used in psoriasis improve localized cutaneous symptoms, but barely affect the underlying causes of the disease [96]. As for what happens with conventional systemic therapies, according to the literature, they can aggravate different factors of the metabolic syndrome [97]. However, a beneficial association with cardiovascular risk has been described with treatment with MTX [98,99,100,101,102]. Different studies attribute protective effects at the vascular level: improvement of endothelial function comparable to that obtained with anti-TNFα [98], reduction in lipoprotein A and serum levels of E-selectin, control of arterial hardening and prevention of elevation of systolic blood pressure [101], a 21% reduction in cardiovascular risk, an 18% reduction in the risk of myocardial infarction and an inverse relationship with stroke [101]. However, none of the above data come from psoriatic patients, but other immune-mediated inflammatory diseases, mainly rheumatoid arthritis. Few studies have evaluated the impact of methotrexate on cardiovascular risk in patients with psoriasis or psoriatic arthritis [102]. A limited influence on platelet function, resistance to insulin and lipid levels, and significant changes in inflammatory biomarkers such as CRP, TNFα, or IL-6 has been observed with the use of MTX in psoriasis patients [101].

With the introduction of biological therapies, expectations of control over cardiovascular comorbidity have increased markedly [43]: data from retrospective studies support the notion that biological drugs may have a cardioprotective effect, reducing the probability that patients with psoriasis will develop CVD [4,103,104,105,106,107], although their role in vascular damage processes remains controversial, probably due to the inconsistency of clinical data on its efficacy against increased cardiovascular risk. Lebwohl (2017) cited the lack of a sufficiently specific and sensitive technology as one of the reasons for this inconsistency [108].

The biological drugs used for psoriasis are mostly monoclonal antibodies that act on specific inflammatory pathways and are administered subcutaneously (with the exception of infliximab, which is administered intravenously) on different weekly schedules. Currently, the biological drugs included in the therapeutic regimen for psoriasis target two key pathways in the development and chronicity of the disease: TNFα signaling and the IL-23/Th17 axis [109,110,111,112].

### 3.1. Anti-TNFα Drugs

TNFα inhibitors have been on the market the longest and are therefore considered first-generation biological drugs. Currently, there are four drugs in this group for the treatment of psoriasis: adalimumab (ADA), etanercept (ETA), infliximab (INF) and certolizumab (CER) [113].

The cardioprotective benefits of anti-TNFα drugs were first documented in patients with rheumatoid arthritis, where they were associated with improved endothelial function [114] and a reduction in all CVDs (RR in rheumatoid arthritis patients treated with TNF inhibitors: 0.70; 95% CI 0.54 to 0.90; *p* = 0.005) [98,107]. The ubiquitous involvement of TNFα in a wide variety of vascular inflammatory responses and its early appearance in the pathogenic cascade have led to the consideration of anti-TNFα therapy as the most promising for controlling cardiovascular comorbidity [43]. Blockade of this cytokine decreases differentiation of T cells to Th1, Th17 and Th22, the subsequent release of IL-17A, IL-17F and IL-22 and the levels of acute phase reactants such as CRP and vascular endothelial growth factor [43]. Although adalimumab and infliximab are contraindicated in patients with moderate–severe heart failure due to several conflicting case reports [115], in patients with psoriasis they seem to improve endothelial function [116], and reduce the risk of myocardial infarction [117,118]. In this sense, a meta-analysis, including 49,795 patients with plaque psoriasis or psoriatic arthritis, confirmed the efficacy of anti-TNFα drugs in decreasing the incidence of cardiovascular events compared with topical/phototherapy or MTX treatment (RR, 0.58; 95% CI, 0.43 to 0.77; *p* < 0.001; I^2^ = 66.2%; RR, 0.67; 95% CI, 0.52 to 0.88; *p* = 0.003; I^2^ = 9.3%; overall: RR, 0.60; 95% CI, 0.48 to 0.74; *p* < 0.001; I^2^ = 57.3%) [117]. In particular, this study demonstrated a decrease in myocardial infarction rate (compared with topical/photo therapy: RR, 0.73; 95% CI, 0.59 to 0.90; *p* = 0.003; I^2^ = 56.2%; compared with methotrexate: RR, 0.65; 95% CI, 0.48 to 0.89; *p* = 0.007; I^2^ = 0.0%) and a trend of lower incidence of mortality compared with control group topical/photo therapy or MTX group (RR, 0.90; 95% CI, 0.54 to 1.50; *p* = 0.68; I^2^ = 70.9%). A retrospective study published later found that, although the rate of myocardial infarction was significantly lower in patients treated with TNFα inhibitors or methotrexate than in those treated with topical treatments, there was no statistical difference when comparing patients using TNFα inhibitors with those treated with methotrexate. Interestingly, when categorizing anti-TNFα patients according to the PASI score response, there were striking differences between PASI 75 (which were considered responders) and PASI 50 (non-responders): the standardized myocardial infarction rate in the group of patients showing PASI 75 improvement after one year of TNF-α inhibitors treatment was much lower than in PASI 50 patients [118]. Although some studies confirm that the risk of CV events using TNF-α inhibitors is lower than with methotrexate treatment [119,120], others report no significant difference in the incidence of MACEs when comparing biologic therapy with conventional treatments or placebo (OR: 1.45, 95% CI: 0.34–6.24) [121]. Therefore, the evidence in psoriasis still remains scarce and more studies are needed to draw definite conclusions [122].

Regarding vascular inflammation, several studies did not report a significant reduction in patients treated with adalimumab [123,124]. One of the latest studies looking at the effect of adalimumab on vascular inflammation and cardiovascular biomarkers confirmed that, when compared to phototherapy, it reduced key markers of inflammation, but with no effect on glucose metabolism and vascular inflammation, or on possible adverse effects associated with high-density lipoproteins [125]. Moreover, a prospective, randomized, controlled head-to-head trial comparing adalimumab and fumaric acid esters on cardiovascular disease markers concluded that adalimumab improved endothelial dysfunction as measured by flow-mediated dilation and showed anti-inflammatory effects by significantly decreasing hsCRP levels; unexpectedly, several parameters of cholesterol metabolism improved in the fumaric acid esters group [126].

Thus, although the literature remains heterogeneous, in part due to methodological differences, anti-TNFα agents have been shown to exert beneficial effects at the cardiovascular level and are beginning to be considered as the preferred systemic agents for the treatment of psoriasis in patients with coexisting cardiovascular risk factors [124], although their mechanisms of action on vascular inflammation remain unknown. A study published in 2020 suggested as part of this mechanism of action that this group of biological drugs could be inhibiting the expression of adhesion molecules, which would hinder leukocyte activation, thereby reducing vascular inflammation [127]. Recent systematic reviews did not show a significant effect of TNF-α inhibitors on subclinical indicators of atherosclerosis in psoriasis or other chronic inflammatory diseases (including as indicators arterial stiffness, carotid intima–media thickness, endothelial dysfunction measured as flow mediated dilation or forearm blood flow and aortic vascular inflammation) [128,129], but did not rule out a positive effect on clinical cardiovascular disease through other pathways, such as primary disease remission or the reduction in the prothrombotic tendency [128].

### 3.2. Drugs That Target the IL-23/Th17 Axis

As has already been described, the cytokine IL-23 is key in the pathogenesis of psoriasis for the differentiation and proliferation of Th17 and Th22. IL-17 released by Th17 lymphocytes is highly relevant in the development of lesions. Currently, three monoclonal antibodies directed against IL-17 are available: secukinumab and ixekizumab, which block IL-17A; and brodalumab, which targets the IL-17A receptor [109,113].

The IL-23 cytokine is a dimer composed of two subunits: p40 and p19, both of which are key to its inhibition. In addition, p40 is not exclusive to IL-23, but rather is shared with IL-12, a cytokine involved in the differentiation of naïve T cells into Th1, therefore drugs directed against this subunit will block both Th17 activation and Th22, as well as Th1. The currently available IL-23 inhibitors are: ustekinumab, whose target is the p40 subunit and which inhibits both IL-23 and IL-12, and three monoclonal antibodies directed against the p19 subunit: guselkumab, tildrakizumab, and risankizumab, that neutralize the activity of IL-23 without affecting IL-12 [109,113].

#### 3.2.1. Anti-IL-17 Drugs

Positive cardiovascular effects are being increasingly documented with anti-IL17A therapy, results which were less expected and more surprising than those obtained with the inhibition of TNFα [130]. It has been described that this cytokine can exert pro-atherogenic [130], inflammatory and prothrombotic effects, destabilizing the atherosclerotic plaque and increasing cell attractants [131], and be related to coronary heart disease [132,133]. However, its role in atherosclerosis remains controversial: depending on the tissue, the cell types involved and the immunological context, antiatherogenic actions are also attributed to this cytokine [130,134]. Moreover, its presence in serum is related to a lower risk of major cardiovascular events [130,135]. These marked differences in the atherogenic role of IL-17 can be explained, at least partially, by differences in the study design, the methods used to inhibit IL-17A, the animal model, and the site of the atherosclerotic lesion, and still remain to be clarified [130].

On the other hand, experimental data suggest that this cytokine could play a central role in the relationship between psoriasis and cardiovascular disease [130], but the existing evidence evaluating the effects of IL-17 inhibitors on the risk of CVD in patients with psoriasis are insufficient [122]. The leading role of neutrophils in vascular inflammation associated with psoriasis may also be linked to the relevance of this cytokine in said process, since neutrophils are the main source of IL-17A in psoriatic skin lesions [8]. In fact, one of the early therapeutic effects of psoriasis treatment with secukinumab is the almost complete elimination of intraepidermal IL-17-positive neutrophils [80]. It has also been shown that the blockade of IL-17A with the biological agent secukinumab improves markers of vascular inflammation in patients with moderate to severe psoriasis, and significantly decreases the prothrombotic environment in an animal model of psoriasis, which supports the hypothesis that this cytokine has a notable role in the vascular system and that it is one of the keys to the development of CVD from psoriasis [127]. The results of the CARIMA study, which evaluates cardiovascular risk markers in patients with moderate to severe plaque psoriasis treated with secukinumab for 1 year, follow the same line: this study concludes that secukinumab treatment can result in improved endothelial function at 52 weeks without proatherogenic changes in the vessel wall or alterations in cardiovascular biomarkers [130]. Despite the fact that, in a recent clinical trial, secukinumab showed a neutral impact on vascular inflammation [136], different studies support the idea that this treatment reduces the cardiovascular impact of inflammation [137] and improves vascular and myocardial function compared with conventional systemic therapies [138]. Despite the presence of baseline cardiovascular risk factors, the risk of MACEs in patients with moderate to severe plaque psoriasis does not seem to increase in the short term [121] or over time—as demonstrated in the most recent update on integrated pooled clinical trial and post-marketing surveillance data on long-term safety of secukinumab over five years [139]—and seem to be comparable to other treatments such as etanercept, as proved by a pooled analysis of 10 phase II and III clinical studies [140]. Notably, in this last study, all documented MACEs occurred in patients with a history of previous or active CVD or risk factors [140,141], so even though treatment with secukinumab should, theoretically, reduce MACE/CVD, we need more long term trials to see whether this is an actuality. As for the other anti-IL17 drugs, ixekizumab and brodalumab have not been shown to have a substantial effect in reducing parameters of cardiovascular impact [35,142].

#### 3.2.2. Anti-IL12/23 Drugs

Anti-IL12/23 agents are the ones that raise the more doubts in terms of cardiovascular impact: although IL-12 and IL-23 have been suggested as potential proatherogenic factors [43,143,144] and, therefore, their inhibition could confer some vascular protection, in most clinical studies the data suggest a neutral profile on cardiovascular events [114,121,145,146]. The initial clinical trials with briakinumab showed a higher number of cardiovascular events [147,148], which prompted monitoring of cardiovascular risk factors in patients in whose dermatological treatment was started with any anti-IL12/23 drug [149], but later, in more advanced trials, no significant differences were found in the number of MACE in individuals treated with this type of biological drug compared to the placebo group [150]. Two different meta-analyses compared the effects of the use of anti-IL-12/IL-23 (ustekinumab and briakinumab) with placebo. The meta-analysis carried out by Ryan et al. [147] included 22 RCTs comprising 10,183 patients and concluded that there was no significant difference in the rate of MACE between patients receiving anti-IL-12/IL-23 and placebo (using the Mantel–Haenszel fixed-effects statistical method: 0.012 events/person-year; 95% CI: −0.001 to 0.026; *p* = 0.12). However, a second meta-analysis including nine RCTs (with a total of 4653 patients) which used a different statistical technique (the Peto one-step method), yielded opposite results and concluded a higher risk of MACEs in patients treated with anti-IL-12/IL-23 drugs (OR = 4.23, 95% CI: 1.07–16.75; *p* = 0.04) [149].

In this same line, a recent case-control study also identified a significant association between the onset of ustekinumab treatment and early occurrence of severe cardiovascular events (OR: 4.17; 95% CI: 1.19–14.59), mainly among patients with high cardiovascular risk [151]. However, longer-term pharmacovigilance studies have, again, not associated its use with such increased risk: the analysis of 3117 patients who received one or more doses of ustekinumab, with 1482 patients treated for 4 or more years (including 838 patients treated for 5 or more years), did not identify an increased risk of MACE (the overall rate of MACE in ustekinumab-treated patients was 0.44/100 patient-year, comparable with those reported for anti-TNF agents in psoriasis) [152]. A second safety surveillance study (the Psoriasis Longitudinal Assessment and Registry (PSOLAR)) which included a higher number of patients (12,093 patients) confirmed these results [153]. Moreover, it has been observed that ustekinumab manages to reduce systemic and vascular inflammation [154] and it has been associated with improvement of coronary artery plaque lipid-rich necrotic core compared with the non-biologic therapy group, and demonstrating no difference with other biological treatments such as anti-TNFα and anti-IL17 drugs [155].

Regarding the monoclonal antibodies directed against the p19 subunit, phase 3 reSURFACE 1 and reSURFACE 2 trials analyzing the efficacy and safety of tildrakizumab in adult patients with moderate to severe chronic plaque psoriasis showed limited changes in cardiometabolic risk factors with no increased short-term risk of CV events [156,157,158]. Similarly, low rates of MACE occurrence have been reported for guselkumab [159,160] and risankizumab [161], however, more studies are still needed to confirm the effect of the anti-IL-23p19 agents on cardiovascular risk.

In general, most data in the literature suggest that anti-IL12/23 have a neutral profile on the development of events and parameters but do not draw definitive conclusions.

A summary of the main studies on the rates of MACEs in psoriatic patients treated with biological agents can be found in Table 1. Unambiguously, there are additional pharmaceutical therapies with potential cardioprotective actions in patients with psoriasis [162].

## 4. Conclusions

There is an association between psoriasis and a higher incidence of CVD, especially in patients with moderate to severe psoriasis, whose relative risk of suffering CVD is increased in 25%, and that of suffering a MACE in 6.2% within 10 years of diagnosis, regardless of traditional risk factors [31,32,33,34,35,36]. Thus, awareness of the need for early detection of subclinical cardiovascular disease and cardiovascular risk factors in psoriatic patients has increased, as well as the necessity to take this into account when choosing the most appropriate therapy.

Psoriasis seems to coexist with a state of vascular inflammation in which circulating neutrophils could be the main cell type involved, and which could explain, at least in part, this increased CV risk. The main biological therapies—which are effective against the skin manifestations of the disease—are not equally effective in controlling this vascular inflammatory environment. In this sense, the anti-TNFα and anti-IL17 drugs appear to be the most advantageous, since many of the studies with adalimumab and secukinumab speak of a general reduction in all CVDs and suggest that their effects go back to the earliest stages of vascular inflammation. In addition, anti-TNFα and anti-IL17 have widely shown better tolerability and fewer side effects than conventional treatments. This should be a compelling reason to rethink the position of these drugs in the general algorithm of treatment of moderate to severe psoriasis, who are at high risk of developing CVDs.

## Figures and Tables

**Table 1:** Summary of the main studies on the rates of MACEs in psoriatic patients treated with biological agents.

Drug	N of Trials or Patients	MACE Risk
Anti-TNFα	5 studies (49795 patients with psoriasis or psoriatic arthritis, mean duration follow-up: 38 months) comparing TNFi (adalimumab, etanercept, golimumab, and infliximab) vs. topical/photo therapy or MTX [117]	vs. topical/photo therapy: RR, 0.58; 95% CI, 0.43 to 0.77; *p* < 0.001; I^2^ = 66.2% vs. MTX: RR, 0.67; 95% CI, 0.52 to 0.88; *p* = 0.003; I^2^ = 9.3% overall, vs. control group: RR, 0.60; 95% CI, 0.48 to 0.74; *p* < 0.001; I^2^ = 57.3%
	4762 psoriasis patients (1058 patients treated with TNFi (adalimumab, etanercept, infliximab); 1331 treated with MTX; 2372 treated with topical agents); a median of 3.9 years follow-up [118]	IR per 1000 SY (95% CI): TNFi: 4.88 (2.5–7.2) (significantly lower than topical cohort, *p* = 0.01) MTX: 5.38 (3.04–8.3) (significantly lower than topical cohort, *p* = 0.02) Topical: 12.34 (9.6–20.8) No significant difference between TNFi and MTX
	11410 TNFi vs. 12433 phototherapy patients [119]	HR = 0.77; *p* < 0.05
	9148 TNFi users vs. 8581 MTX users [120]	HR = 0.55; *p* < 0.01
	18 RCTs comparing TNFi (4 adalimumab, 9 etanercept, 5 infliximab) vs. placebo [121]	OR, 0.67 (95% CI, 0.10–4.63, *p* = 0.69)
	15 RCTs comparing TNFi (8 etanercept, 4 infliximab, 3 adalimumab) vs. placebo [147]	(Mantel–Haenszel risk difference, −0.0005/SY; 95% CI, −0.010 to 0.009; *p* = 0.94)
Anti-IL17 (Secukinumab)	28 clinical trials and post-marketing surveillance data, 12,637 patients (15063, 5985 and 3527 patient-years of exposure in psoriasis, psoriatic arthritis and ankylosing spondylitis patients, respectively); 5 years cumulative data [139]	IR < 0.4/100 SY for psoriasis and psoriatic arthritis, with no apparent increases over time
	10 phase II/III clinical trials, 52 weeks follow-up [140]	IR: 0.42/100 SY (300 mg dose) IR: 0.35/100 SY (150 mg dose)
Anti-IL12/23	7 RCTs (ustekinumab vs. placebo), 30 weeks follow-up [121]	OR, 4.48 (95% CI, 0.24–84.77; *p* = 0.32)
	1582 ustekinumab vs. 732 placebo-treated patients. 20 weeks follow-up [146]	IR, 0.3%; (95% CI, 0.1–0.70) vs. 0.0% (95% CI, 0.0–0.5%)
	9 RCTs comparing anti-IL-12/23 (5 ustekinumab, 4 briakinumab) vs. placebo [147]	(Mantel–Haenszel risk difference, 0.012/SY; 95% CI, −0.001 to 0.026; *p* = 0.12
	9 RCTs (ustekinumab vs. placebo), 30 weeks follow-up [149]	OR, 4.23, 95% CI: 1.07–16.75, *p* = 0.04)
	Case-time-control study with 9290 ustekinumab [151]	Patients with high CV risk: OR, 4.17; 95% CI, 1.19–14.59 Patients with low CV risk: OR, 0.30; 95% CI, 0.03–3.13
	4 phase II and phase III studies, 3177 patients, 5 years follow-up [152]	IR (45 mg) 0.56/100 SY IR (90 mg) 0.36/100 SY
	Psoriasis Longitudinal Assessment and Registry (PSOLAR), 12093 patients [153]	IR 0.33/100 SY
	1465 patients (981 briakinumab vs. 484 placebo) [148]	Exposure-adjusted rate: 1.06/100SY, 95% CI 0.43, 2.18.
	9 RCTs (briakinumab vs. placebo), 30 weeks follow-up [149]	OR, 4.47 (95% CI, 0.69–28.89; *p* = 0.12)

CI: confidence interval; HR: hazard ratio; IR: incidence rate; MTX: methotrexate, SY: subject-years of exposure, OR: odds ratio; RCTs: randomized controlled trials; TNFi: TNFα inhibitors.

## Data Availability

Data sharing not applicable.
